# Latin American Study of Nutrition and Health (ELANS): rationale and study design

**DOI:** 10.1186/s12889-016-2765-y

**Published:** 2016-01-30

**Authors:** M. Fisberg, I. Kovalskys, G. Gómez, A. Rigotti, L. Y. Cortés, M. Herrera-Cuenca, M. C. Yépez, R. G. Pareja, V. Guajardo, I. Z. Zimberg, A. D. P. Chiavegatto Filho, M. Pratt, B. Koletzko, K. L. Tucker, the ELANS Study Group

**Affiliations:** 1 Instituto Pensi, Fundação Jose Luiz Egydio Setubal, Hospital Infantil Sabara, São Paulo, 01239-040 Brazil; 2 Universidade Federal de São Paulo, São Paulo, 04023-062 Brazil; 3 Commitee of Nutrition and Wellbeing, International Life Science Institute (ILSI-Argentina), Buenos Aires, C1059ABF Argentina; 4 Departamento Nutricion, Facultad de Ciencias Medicas, Universidad Favaloro, Buenos Aires, C1078AAI Argentina; 5 Departamento de Bioquímica, Escuela de Medicina, Universidad de Costa Rica, San José, 11501 Costa Rica; 6 Departamento de Nutrición, Diabetes y Metabolismo, Centro de Nutrición Molecular y Enfermedades Crónicas, Escuela de Medicina, Pontificia Universidad Católica, Santiago, 833-0024 Chile; 7 Departamento de Nutrición y Bioquímica, Pontificia Universidad Javeriana, Bogotá, Colombia; 8 Centro de Estudios del Desarrollo, Universidad Central de Venezuela (CENDES-UCV)/Fundación Bengoa, Caracas, 1010 Venezuela; 9 Colegio de Ciencias de la Salud, Universidad San Francisco de Quito, Quito, 17-1200-841 Ecuador; 10 Instituto de Investigación Nutricional, Lima, 15026 Peru; 11 Departamento de Epidemiologia, Faculdade de Saúde Pública, Universidade de São Paulo, São Paulo, 01255-000 Brazil; 12 Nutrition and Health Sciences Program, Hubert Department of Global Health, Rollins School of Public Health, Emory University, Atlanta, 30322 USA; 13 Division of Metabolic and Nutritional Medicine, Dr. von Hauner Children’s Hospital, University of Munich Medical Center, D-80337 Munich, Germany; 14 Department of Clinical Laboratory and Nutritional Sciences, University of Massachusetts Lowell, Lowell, 01854 USA; 15 Rua Borges Lagoa, 1080, Vila Clementino, São Paulo CEP 04038-002 Brazil

**Keywords:** Nutrition, Physical activity, Latin America, Cross-sectional study

## Abstract

**Background:**

Obesity is growing at an alarming rate in Latin America. Lifestyle behaviours such as physical activity and dietary intake have been largely associated with obesity in many countries; however studies that combine nutrition and physical activity assessment in representative samples of Latin American countries are lacking. The aim of this study is to present the design rationale of the Latin American Study of Nutrition and Health/*Estudio Latinoamericano de Nutrición y Salud* (ELANS) with a particular focus on its quality control procedures and recruitment processes.

**Methods/Design:**

The ELANS is a multicenter cross-sectional nutrition and health surveillance study of a nationally representative sample of urban populations from eight Latin American countries (Argentina, Brazil, Chile, Colombia, Costa Rica, Ecuador, Perú and Venezuela). A standard study protocol was designed to evaluate the nutritional intakes, physical activity levels, and anthropometric measurements of 9000 enrolled participants. The study was based on a complex, multistage sample design and the sample was stratified by gender, age (15 to 65 years old) and socioeconomic level. A small-scale pilot study was performed in each country to test the procedures and tools.

**Discussion:**

This study will provide valuable information and a unique dataset regarding Latin America that will enable cross-country comparisons of nutritional statuses that focus on energy and macro- and micronutrient intakes, food patterns, and energy expenditure.

**Trial Registration:**

Clinical Trials NCT02226627

## Background

According to the World Health Organization, more than 1.4 billion adults were overweight and more than half a billion were obese worldwide in 2008, and the prevalence of obesity nearly doubled between 1980 and 2008 [1]. According to World Health Organization, in the Americas including the United States and non-continental countries, 61 % of adults are overweight or obese in 2014 [2]. In Latin America, nearly a quarter of the population is obese, and the prevalence has increased to a greater magnitude in Mexico, Argentina, and Chile. A recent review estimated that 20–25 % of the children and adolescents (0–18 years) of Latin American are overweight or obese [3]. Time trends suggest that these figures might rise further and by 2030 up to 81.9 % of the Latin American and the Caribbean adult population could be either overweight or obese [4].

These rapid epidemiological changes in the majority of developing countries over the last decades have occurred in the framework of the so-called nutritional transition. The concept of the nutritional transition includes a shift in dietary intake and energy expenditure both of which are influenced by ongoing interactions between economic, demographic, environmental, psychosocial and cultural factors and are occurring simultaneously in society [5]. Latin American countries are experiencing different stages of the nutrition transition, although the prevalence of undernutrition is declining at different rates, and the prevalence of overweight is dramatically increasing [6].

Eating patterns that are characterized by high energy density diets, increased intakes of processed foods containing large amounts of refined sugars and saturated fats, and low intakes of fiber and micronutrients is considered the major preventable behavioral risk factors for obesity [7–9]. Physical inactivity and sedentary behavior are also a preventable behavior associated with obesity, but the evidence on this issue remains mixed, [10], and the results might not be generalizable to all world regions [11]. Furthermore, most of the information available about the role of dietary and physical activity profiles in obesity has come from studies that have been conducted in high income, developed countries, but similar evidence from developing countries is limited. In the last decade studies started to be conducted in developing countries such as Brazil [12] and China [13]. However, there is insufficient evidence about whether the same dietary and physical activity patterns and similar associations with various sociodemographic conditions can be found in different regions of middle-income countries.

Although national surveys on nutrition already exist in some Latin American countries [14–21], the majority of the data available are based on Household Consumption and Expenditures Surveys which provide consumption data at the household level and are not accurate for calculations of individual energy intake, or representative samples of entire population in each country were not employed.

An overview of the most recent nutrition surveys conducted at individual level in nationally representative household samples of Latin America is presented in Table 1. Only a handful of countries, however, have conducted surveys with face-to-face assessment of food intake for reasons of cost, expediency and logistics [22].

**Table 1:** Cross-sectional household nutrition surveys conducted in representative samples of Latin America

Country	Year of data collection	Sample size	Sample size that underwent dietary assessment	Method	Analysis of the dietary data	Reference
Argentina (National Survey of Nutrition and Health - ENNyS)	2004–2005	36,354 (aged 6 m - 5 y and women 10–49 y)	36,354	24-h Recall	Food Composition database developed for ENNyS	[16]
Brasil (Household Budget Survey - POF)	2008–2009	159,941 (aged ≥ 0 y)	34,003 (aged ≥ 10 y)	Two 24-h recall	NDSR software [44] and Food Composition database developed for POF [45]	[46]
Colombia (National Nutritional Situation Survey -ENSIN)	2008–2010	162,331 (aged 0–64 y)	17,897 (aged 5–64 y)	Food-Frequency Questionnaire	Qualitative (daily frequency of intake)	[18]
Chile (National Food Consumption Survey - ENCA)	2014	4920 (aged ≥2 y)	4920	Quantitative Food-Frequency Questionnaire and 24-h Recall	PC-SIDE software	[14]
Ecuador (Ecuadorian National Health and Nutrition Survey - ENSANUT-ECU)	2011–2013	57,727 (aged 0–59 y)	19,932 (aged 1–59 y)	24-h Recall	PC-SIDE software	[47]
México (National Health and Nutrition Survey - ENSANUT)	2012	96,031 (aged >0 y)	10,563 to 12,484 according to method used	Semi-quantitative Food Frequency and 24-h recall in 11 % and 13 % of sample, respectively	Food Composition database developed by National Institute of Public Health [48]	[49]
Perú (National Survey of Nutritional, Biochemical, Socioeconomic and Cultural Indicators – ENINBSC)	2006	4206 (aged ≥20 y)	4206	24-h Recall	ANDREA software [50], developed by CENAN-INS	[20]
Venezuela (Encuesta de Seguimiento al Consumo de Alimentos - ESCA)	2012-2014	20,670 (aged ≥ 3 y)	6316 participants aged ≥ 3 y	Diet history and food frequency questionnaire	Food Composition database developed for ESCA	[51]

Determinations of the dietary patterns and energy and nutrient intakes are critical for developing dietary recommendations and policies to address the adverse consequences of inappropriate dietary patterns and physical inactivity. This information would be of the greatest actionable value to governments, the food and beverage industries and agriculture.

Studies that combine nutrition and physical activity assessment in representative samples of Latin American countries are lacking. In this direction, the Latin American Study of Nutrition and Health/*Estudio Latinoamericano de Nutrición y Salud* (ELANS), which is randomized cross-sectional multicenter investigation of the nutritional and physical activity statuses of adolescents and adults in eight Latin American countries, was designed. The study aims to (1) provide up-to-date reliable and comparable data of dietary intake, physical activity, and its association with anthropometric profile among representative urban populations of eight Latin American countries; (2) measure variation in overweight, dietary intake and physical activity by region, cultural background, socioeconomic status, age and gender; (3) add new scientific-based evidence to describe the interplay among energy intake, energy expenditure, and anthropometric measurements. Our overarching hypothesis is that the relationships between dietary and physical activity profiles will differ across countries and across different environmental settings.

Up to now, there is no Latin American study using a central standard methodology across a group of participating countries. Previous multicenter studies [23–26] were important references to determine the rationale and design of ELANS.

The purpose of this report is to describe the design and methodology of the ELANS. The study is currently ending the fieldwork data collection.

### Overall design and methods

**Fig. 1:**
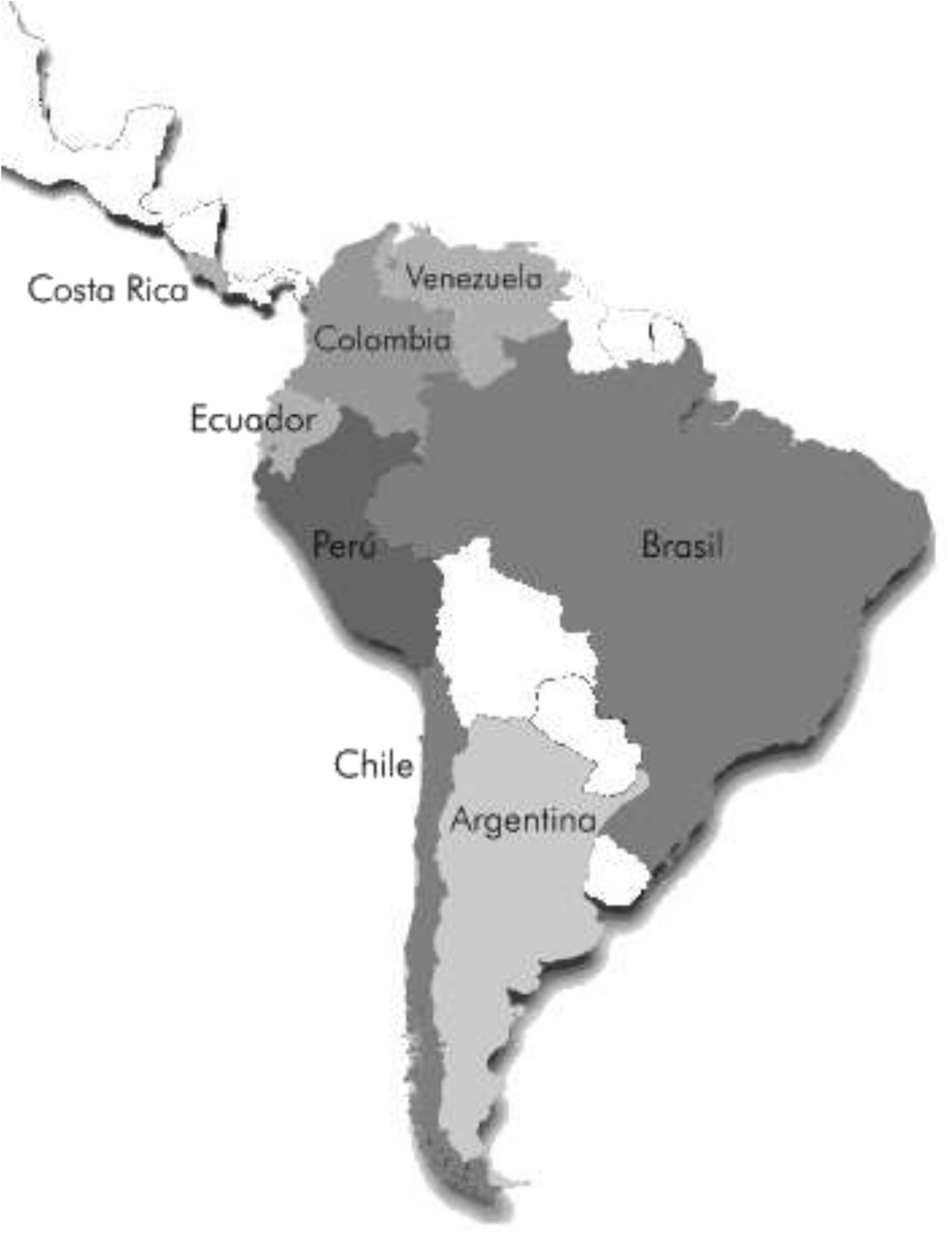
Latin American countries included in ELANS

The ELANS is a household-based multi-national cross-sectional survey that was conducted over a period of one year in eight Latin American countries (i.e., Argentina, Brazil, Chile, Colombia, Costa Rica, Ecuador, Perú, and Venezuela; Fig. 1) that represent approximately 60 % of the total countries of Latin America. All of the study sites are academy (universities and other academic institutions)-based and adhered to a common study protocol for interviewer training, implementation of fieldwork, data collection and management, and quality control procedures that will be simultaneously performed.

### Sampling procedures

A random complex, multistage sampling of 9000 adolescents and adults aged 15.0–65.0 years, stratified by geographical location (only urban areas), gender, age and socioeconomic status was performed to select a representative sample of the urban household population of eight Latin American countries. Only urban areas were included in order to keep homogeneous population in the research, and based on the fact that almost all countries have at least 80–90 % of their population living in urban areas. Individual quotas were defined for each of these variables, which allow the identification of the total numbers of interviews required to properly represent the socio-demographic distribution under study. The survey was designed in order that no more than one subject were selected from a household.

The sampling size was calculated with a confidence level of 95 % and a maximum error of 3.49 %. A survey design effect of 1.75 was estimated based on guidance from the U.S. National Center for Health Statistics [27], and calculations of the minimum sample sizes required per strata (i.e., socioeconomic level (SEL), age, and gender) were performed for each country. Table 2 shows a detailed description of the case numbers required per strata in each of the 8 study sites.

**Table 2:** Estimated distributions of country-specific samples according to age range, gender, and SEL

	Age range				Gender		SEL			Total sample size	Margin of error (%)
	15–19.9 years	20–34.9 years	35–49.9 years	50–65 years	Male	Female	High	Middle	Low		
Argentina	144	444	336	276	576	624	72	504	624	1200	2.83
Brazil	220	760	600	400	940	1060	520	940	540	2000	2.19
Chile	113	305	270	183	426	444	61	348	461	870	3.32
Colombia	148	443	357	283	615	615	62	357	812	1230	2.79
Costa Rica	111	300	229	150	395	395	87	450	253	790	3.49
Ecuador	128	312	224	136	400	400	104	576	120	800	3.46
Peru	165	440	308	187	528	572	209	374	517	1100	2.95
Venezuela	143	418	319	220	528	572	55	154	891	1100	2.95

The demographic data were obtained from the appropriate national statistics institutes of each country. The selection of the households was design in four stages. In the first stage, total urban population was employed to define main regions proportionally in each country first, and then select cities representing each region - main cities and other cities representatives of the region, mixing a random method and sampling criteria, and trying to fulfill urban population coverage to the maximum possible. This mixed criterion enabled an increase in the efficiency of the fieldwork according to the study characteristics. In the second stage, sampling points (census tracts) of each city were randomly selected. In the third stage, a cluster of households was selected from each sampling unit. Addresses were chosen systematically using standard random route procedures, beginning with an initial address selected at random and selection of households with three systematic jumps, that is, the selection of a given household was made by randomly picking the first home and subsequently skipping 3 households. Finally, in the fourth stage, selection of respondent within a household was performed using two criteria: in each sampling unit, half of the households the participant was selected by the next birthday criteria; in the other half selection was by quotas of gender, age, and SEL.

The field interviewer arrived at the home, show an official identification badge, a letter introducing ELANs study, and briefly explain the survey’s purpose. A screener questionnaire to enumerate the household and to determine eligibility to participate further in ELANS was fulfilled. Thereafter, the selected participant signed the informed consent form.

A telephone number was available for the participants in order to answer any questions about the research, and use of the accelerometer device.

In the following circumstances - unsuccessful attempts to contact the target individual, total refusal to participate, obstruction by a family member, or inability to participate for a specified reason (e.g., travel, agenda, hospitalization) - substitutes were chosen in the home next door, following the same random selection criteria described above.

As exclusion criteria were considered pregnant and lactating women (in the first 6 months postpartum), individuals with major physical or mental impairments that affect food intake and physical activity (e.g., musculoskeletal disease, recent surgery, severe asthma, dementia, major depression), individuals below 15 or over 65 years old, adolescents without assent and consent of a parent or legal guardian, individuals living in any residential setting other than a household (e.g., hospitals, regiments, and nursing homes), and individuals unable to read.

### Ethical issues

The overarching ELANS protocol was approved by the Western Institutional Review Board (#20140605) and is registered at Clinical Trials (#NCT02226627). Each site-specific protocol was also approved by the ethical review boards of the participating institutions. All participants provided informed consent/assent for participation in their country-level study. Participant confidentiality for the pooled data is maintained via the use of numeric identification codes rather than names. All data transfer was done with a secure file sharing system.

### Data collection instruments

**Fig. 2:**
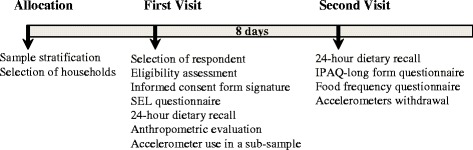
Study design

The ELANS protocol includes data collected via questionnaires and objective measurements. The questionnaires were administered in 2 household visits (Fig. 2). The first visit involved the selection of a respondent within the household. Additionally, the first visit also included application of the SEL questionnaire and 24-h dietary recall (24-h), and assessment of anthropometric measurements. In the first visit, a subsample received instructions regarding the use of an accelerometer with a diary to be filled out for 7 consecutive days. The second visit was performed 8 days after the first contact for the participants who were given accelerometers and 4 days later for the participants who were not given accelerometers. The second visit also included the administration of a second 24-h, the IPAQ-Long Questionnaire, and a beverage intake questionnaire (BEVQ) and the retrieval of the accelerometer.

### Dietary assessment

The 24-h method was selected because of its nearly universal applicability across populations with varying literacy skills and its relatively low burden for participants [28]. Each recall was conducted using the Multiple Pass Method [29] and ascertained all foods, nonalcoholic and alcoholic beverages, water and dietary supplements consumed over the prior 24 h in depth. The BEVQ was designed to obtain the frequency of beverage intake across 10 beverage categories (water, flavored water, soft drinks, fruit drinks, sport drinks, energy drinks, tea and coffee drinks, other non-alcoholic drinks, and alcoholic drinks). For each beverage, participants answered whether they consume the specific category of beverage, the frequency of intake (daily, weekly, monthly), and how often they drink the beverage during the selected unit (1–10 occasions). The list of beverages included in the questionnaire was standardized as much as possible across the ELANS countries; however, regional variations in beverage consumption patterns required some cultural and regional adaptations for some items within beverage categories.

The food and beverages intakes recorded with the 24-h is transformed into energy, macronutrients and micronutrients values using the Nutrition Data System for Research version 2013 software (NDS-R, University of Minnesota, MN). A food matching standardized procedure involving nutritional equivalency of local food items reported by the study participants into foods available in NDS-R database was strictly conducted by each country, and describe in detail elsewhere [30]. A concordance rate of at least 80 % to 120 % for energy and macronutrient content was required to establish a nutritional equivalency of local food items to foods available in NDS-R database.

The Multiple Source Method (MSM) was used to estimate the usual intake of each nutrient, food and food groups. The method was developed by researchers at the European Prospective Investigation into Cancer and Nutrition (EPIC) The MSM method is a mixed model comprised of three parts which requires at least two days of short-term dietary measurements (such as 24-h) on a random subsample of the target population. In the first part, the probability of consumption of a food/nutrient on a day is estimated using logistic regression with random effects (probability model). Second, data that has been transformed for normality is used to estimate usual amount of food intake on days of consumption using linear regression, also with random effects (quantity model). In the final part, the individual usual food/nutrient intake is calculated by multiplying the probability of consumption of a food/nutrient (part 1) with the usual amount of food intake (part 2) [31]. The means and percentiles of the intakes will be estimated for each sex. The MSM method is a web-based statistical modeling, available at MSM website (https://msm.dife.de/tps/en).

### Physical activity measures

#### Self-reported activities

Self-reported physical activity was assessed using the International Physical Activity Questionnaire (IPAQ)-long version, a validated self-report measurement tool for physical activity in Latin America [32]. The Mexican (Spanish) version of IPAQ [33] was adapted for all countries of ELANS, using culturally appropriate wording and examples. Only the sections leisure-time and transport physical activity (LTPA and TPA) were included, due to greater importance of these domains in public health and poor validity of the occupational and home-based PA IPAQ sections in Latin American urban settings. These sections are the most relevant for categorizing population levels of physical activity and for guiding public health policies and programs [32].

A domain-specific activity score is calculated separately for each domain of physical activity (transportation and leisure-time). Total times engaged in walking, moderate physical activity and vigorous physical activity, all expressed in min/week, are scored using established methods posted at the IPAQ website (www.ipaq.ki.se).

Additionally, information not included as part of the summary score of physical activity, such as sedentary activities (reading, television viewing and sitting at a desk), will be analyzed.

#### Objectively assessed activity

To objectively monitor physical activity and inactivity, 40 % of the sample was asked to wear a triaxial accelerometer (model GT3X+, ActiGraph, Pensacola, FL, USA) on an elasticized belt at hip level on the right mid-axillary line for 7 days. The participants were asked to wear the device while they are awake and to take it off for sleeping, showering or swimming. Verbal (in person and by demonstration) and written instructions on how to wear the accelerometer were provided. To further ensure protocol compliance, participants filled in an accelerometer log indicating the start- and end-time of use per day. Following the final day of data collection, the accelerometers was returned to the study sites, and the research team verified the data for completeness using the ActiLife software version 6 (ActiGraph, Pensacola, FL). At least 5 days of recording with a minimum of 10 or more hours of registration per day including at least one weekend day were required for data inclusion and analysis. The sampling interval (epoch) was set at 30 records per second.

### Anthropometric measurements

In each country, the anthropometric measurements of body weight, height and waist, hip and neck circumferences were collected according to standardized procedures. The participants were measured after all heavy clothing, pocket items and shoes and socks are removed. Body weight was measured with a calibrated electronic scale up to 200 kg with an accuracy of 0.1 kg. Height was measured with a portable stadiometer up to 205 cm with an accuracy of 0.1 cm. The measurements were taken during inspiration, with the base of the stadiometer lightly touching the upper reaches of the head and with the participant’s head in the Frankfort Plane [34]. The circumferences were measured with an inelastic tape to the nearest 0.1 cm. Waist circumference was measured according to World Health Organization recommendations, i.e., with the participants standing, after a regular expiration, to the nearest cm, midway between the lowest rib and the iliac crest [35]. Hip circumference was recorded at the level of the greatest posterior protuberance of the buttocks, with the tape held horizontally flat without pressing the soft tissues. Neck circumference was measured at the point just below the larynx (thyroid cartilage) and perpendicular to the long axis of the neck (with the tape line in the front of the neck at the same height as the tape line at the back of the neck) [36].

The interviewers were trained to collect all measurements by certified nutritionists/dietitians who will simultaneously operate as supervisors of the fieldwork. Each measurement was repeated twice to ensure accuracy, and the average used for the analyses. If the two readings differ by more than the previously established set point (0.1 kg for weight, 0.5 cm for height, 0.5 cm for neck circumferences, and 1 cm for waist and hip circumferences), then a third measurement was taken. All three measurements were recorded, and the outlier excluded during the data cleaning process.

The body mass index (BMI; weight (kg)/height (m^2^)) and waist-to-hip ratio were calculated. The absolute values of each circumference measurement were compared to predefined cutoff points according to age and gender.

### Demographics and SEL

A questionnaire was used to collect information about demographics such as age, gender, years of education, number of people in the household, race/ethnicity, marital status, and number of years living in the country.

Socioeconomic level was also evaluated by questionnaire using a format that will be country-dependent and based on the legislative requirements or established local standard layouts. SEL data was divided into three strata (high, medium and low) based on the national indexes used in each country [37–43].

### Pilot study

To examine all procedures and verify that the planned activities could be adequately executed, a pilot study was performed on 50 participants from each country 2 months before starting data gathering for the full study protocol. This pre-test included all procedures from the selection of volunteers to the analyses of data consistency. Principal investigators of each country provided information, their opinion and experience with regard to data collection. This pilot study allowed for the fine-tuning of the study protocol, among other things, the pilot test allowed measurement of the time required for the execution of various activities, for example the first and second visit, data transfer from the pollster to the investigator, the review and survey data entry. It also allowed verification of the exactitude of the data collected and identification of the issues that should be emphasized for the second training session for pollsters, prior to the beginning of the study. The results of this process did not reveal the need to implement any changes in the proposed methodology. Due to the lack of security in many cities, it was verified the need for greater identification of interviewers (badges, cover letter, apron lab coat).

### Study management

The management of ELANS was designed to ensure effective collaboration and communication between the eight study centers involved in this cross-sectional study. Investigators from each participating center were involved in the planning and development of the protocol, which included the study design.

Two chairs (from Argentina and Brazil) and a co-chair (from Costa Rica) are responsible for the overall coordination of the study. Each study site is managed by a local principal investigator (PI), who is responsible for all aspects of data collection at the local level.

A database center is responsible for the creation and management of a central database in Argentina. Two researchers in Brazil are responsible for the management of a central database and for the analysis of the accelerometer data. A statistician and his team in Brazil are responsible for the data analysis and the generation of preliminary reports.

To facilitate data collection, entry and management, a secured web-based system was used. Data from each study site entered remotely using a standard File Transfer Protocol (FTP) web-browser, and the system allowed both the study site staff and the coordinating center to monitor the progress and generate missing data reports in real time.

A steering committee of four external advisors with extensive and diverse scientific background was invited to assess the overall progress of the study and to provide guidance regarding the overall study direction and study goals. External advisor had expertise in designing multicentric epidemiological studies, nutrition and physical activity survey, and statistical analysis.

With the exception of requiring that the study be global in nature, the study sponsor had or will have no role in the study design, data collection and analysis, decision to publish, presentation and/or manuscript preparation.

Once the study is complete, the data will be available for sharing with the international scientific community.

### Personnel training

To validate and harmonize the methodologies, a general training meeting was held with all of the PIs and the ELANS coordination center. The scientists in charge of every research tool attended the meeting. At the end of this training, national teams were placed in charge of translating the field protocol into the local language and submitting the protocol to the local ethical review boards of the participating institutions. Each study site was also responsible for training their personnel for the pilot and field studies. The interviewers were required to have completed at secondary education. Training lasted from 7 to 10 days at each site.

### Quality control strategies

Quality control strategies were applied using a framework that comprehensively considered each phase of the study. To ensure accurate, standard and consistent measurements throughout this multicenter study, a variety of procedures were used and are described in Table 3. One the key aspects of the quality control was the development of the manual of operations to keep same methodology and procedures among all sites. The crucial roles of the PIs were focused on supervising of pilot test and fieldwork, identify and decide on the necessary amendments to the study protocol, coordinate all the trainings, ensuring accuracy of data entry and identifying inconsistencies and standardized procedures across the sites. The coordinating center audited the complete procedures for the countries.

**Table 3:** Quality assurance strategies

Levels of quality control	Phases			
	Design and planning	Pilot testing	Data collection	Data analysis
Coordinating center	•Critical review of protocols •Harmonization of manual of operations for eight study sites •Coordination of timelines and activities	•Smoothness and feasibility of field operations assessed	•Monitoring field activities	•Audit and evaluate validity of findings prior to publication •Internal peer reviews prior to publication
Principal Investigators	•Review of design and planning of the study •Regular meetings with coordinating center	•Audit after completion of the pilot	•Supervising and ensuring accuracy of data entry	•Validity checks •Results review
Field Personnel	•Extensive training over a period of 7 to 10 days-theory and practical-by the study managers	•Evaluated all field and documenting difficulties	•Field coordinator will assure that procedures for data collections and quality control are followed •Additional training when necessary	
Survey Questionnaires	•Peer-reviewed •Validated •Translated to local languages	•Consistency in small pilot study will be established	•Regular checks done to assess completeness	•Incomplete questionnaires identified and discarded
Measuring Equipment	•Standardization of equipment and measurements •Acquired by each country •Development of anthropometric procedures manual	•Evaluation of calibration techniques, acceptability of use in field	•Regular calibration of equipment; faulty equipment replaced when required	
Documentation	•Assurance of standardized procedures across the sites •Training in appropriate and legible documentation	•Recording legibility assessed	•Audit recordings	
Data Storage & Confidentiality	•Data back-up and protection policies established	•Accessibility of software assessed	•Identify inconsistencies •Corrective actions •Locked and password protected data storage •Active back-up	•Datasets identified •Access to personal identifiers limited
Data Entry	•Training of staff •Protocols, consistent data cleaning methods and verification systems established	•Variability assessments conducted	•Interim analyses to identify duplicate entries	•Reporting of outliers •Validity checks •Database errors tracked

### Analysis plan

The primary outcome of interest will be to describe the distributions of overweight and obesity, and energy intake and expenditure intake across the countries and regions of the study. A secondary objective of the study will be to identify the individual characteristics associated with the two main outcomes of interest (obesity and energy intake/expenditure) and to test whether these associations vary across the different regions by applying the statistical methodologies described below. These individual characteristics of interest will include, but are not be limited to, education, marital status, age, sex and individual income. The results of the three main outcomes will also be presented after stratification for each of the individual characteristics.

Initially, basic statistical models will be applied to analyze the aggregated results for all the individuals in the multicenter study to identify the individual characteristics (independent of area of residence) associated with each of the outcomes, with the inclusion of all the projected 9000 individuals. Separate individual models for each of the countries will also be performed.

We will also test the presence of statistically significant differences between regions of residence and countries by adjusting multilevel models (also known as hierarquical linear models), where the individual results will be included as the first level of the model and the region of residence as the second level of the model. Overweight and obesity will be analyzed separately as dichotomous dependent variables (vs. normal weight) by adjusting logistic multilevel models, while energy intake and expenditure will be treated as continuous dependent variables and multilevel linear models will be adjusted. We will test for a statistical difference of the dependent variables according to region of residence (a two-level model) and also by including the country of residence as the third level of the previous multilevel models. Statistical difference between the regions and the countries will be tested by analyzing the area-level variance (between-group variation) of each multilevel model.

Another objective will be to identify the pathways that could lead to overweight/obesity and higher energy intake/expenditure. Some of the candidates include lower income leading to higher consumption of nutritionally poor products leading to obesity, marital status leading to higher energy intake, among many other possibilities. These options will be tested by adjusting Structural Equation Models (SEM), where each of the pathways that lead to the dependent variables will be tested independently for statistical significance. We will also calculate the Standardized Root Mean Square Residual (SRMR) for each of the final structural models to assess its goodness-of-fit, with the objective of selecting the models with the pathways that best fit the data. Finally, given the multicentric nature of the sample, we will also fit structural models with clustered standard errors to relax the assumption of independence of observations within clusters of regions and countries.

Further specific sub-projects will depend on the results and associations found for the initial analyses. Some of these sub-projects include analyses of micronutrient ingestion, snack consumption and the determinants of regular physical exercise levels. Other projects will follow depending on the results and associations observed in the main analyses. Some of the individual variables regarding dietary and physical activity patterns will likely be aggregated, depending on their interdependent relationships and previous studies in the literature, by applying factor analysis. The final datasets compiled from each country will be standardized into groups of variables and categories and sent to the coordinating center in Brazil. Descriptive statistical analyses, multilevel analyses, and structural equation models will be performed with R 3.2.0, MLwiN 2.29, and Stata 13, respectively.

## Discussion

The ELANS is a comprehensive cross-sectional multicenter investigation of the nutritional and physical activity statuses of adolescents and adults in 8 Latin American countries.

An important strength of the ELANS study concerns the number of participating countries from different regions in Latin America including countries that lack data on dietary intake and physical activity level of its population. In addition, the data set allows unique comparisons of dietary and physical activity patterns, as all measurements were obtained according to standard methodology and protocols in all participating countries and all countries started the fieldwork in very near time. The objective measures of physical activity with accelerometer from subgroups of respondents, further enriches the data set. Another strength of the study is the selection and adaptation of a food composition database to make cross-country nutritional intake comparisons. Standardization at the food and nutrient levels will likely minimize systematic and random errors in nutrient intake estimations because between-country comparisons are particularly prone to error when different food composition tables are used to estimate dietary intake.

The study also has several potential weaknesses. The ELANS is currently a cross-sectional study with all of the inherent limitations of this type of design. This means that it will be able to explore correlates of dietary intake, physical activity and obesity, but not its causal determinants. One of the issues of the ELANS is the variations in SEL questionnaire between countries since its format was based on the legislative requirements or established local standard layouts. That may reduce the validity of cross-country comparisons. The ELANS countries span a wide range of health, social and economic indicators; however, the results of the study may not be directly generalizable to other countries. Nevertheless, no study has evaluated the nutritional statuses and physical activity patterns of adolescent and adult populations in Latin American using a standardized methodology across a consortium of several participating countries. This study will provide a unique dataset from Latin America that will enable cross-country comparisons of nutritional status that focus on both energy intake and expenditure. The findings of this study should affect the planning of health policies and programs that are designed to control nutritional inadequacies and low levels of physical activity, and their consequences, as well as the local and cultural adaptation of these policies and programs for implementation in Latin American countries.
